# Prevalence and Diversity of Antibiotic Resistant *Escherichia coli* From Anthropogenic-Impacted Larut River

**DOI:** 10.3389/fpubh.2022.794513

**Published:** 2022-03-10

**Authors:** Chui Wei Bong, Kyle Young Low, Lay Ching Chai, Choon Weng Lee

**Affiliations:** ^1^Institute of Biological Sciences, Universiti Malaya, Kuala Lumpur, Malaysia; ^2^Institute of Ocean and Earth Sciences (IOES), Universiti Malaya, Kuala Lumpur, Malaysia; ^3^Institute for Advanced Studies, Universiti Malaya, Kuala Lumpur, Malaysia

**Keywords:** *Escherichia coli*, anthropogenic activities, antibiotic, resistant, resistance genes

## Abstract

Aquatic environments, under frequent anthropogenic pressure, could serve as reservoirs that provide an ideal condition for the acquisition and dissemination of antibiotic resistance genetic determinants. We investigated the prevalence and diversity of antibiotic-resistant *Escherichia coli* by focusing on their genetic diversity, virulence, and resistance genes in anthropogenic-impacted Larut River. The abundance of *E. coli* ranged from (estimated count) Est 1 to 4.7 × 10^5^ (colony-forming units per 100 ml) CFU 100 ml^−1^ to Est 1 to 4.1 × 10^5^ CFU 100 ml^−1^ with phylogenetic group B1 (46.72%), and A (34.39%) being the most predominant. The prevalence of multiple antibiotic resistance phenotypes of *E. coli*, with the presence of *tet* and *sul* resistance genes, was higher in wastewater effluents than in the river waters. These findings suggested that *E. coli* could be an important carrier of the resistance genes in freshwater river environments. The phylogenetic composition of *E. coli* and resistance genes was associated with physicochemical properties and antibiotic residues. These findings indicated that the anthropogenic inputs exerted an effect on the *E. coli* phylogroup composition, diversification of multiple antibiotic resistance phenotypes, and the distribution of resistance genes in the Larut River.

## Introduction

*Escherichia coli* (*E. coli*) of the family *Enterobacteriaceae*, is a Gram-negative, facultative anaerobe, non-spore-forming, rod-shaped, commensal, and potentially pathogenic bacterium that resides largely in the gastrointestinal tracts of warm-blooded vertebrate animals (1–3). Most *E. coli* strains are harmless, and only some are pathogenic. The pathogenic *E. coli* can be classified as either intestinal pathogenic *E. coli* (IPEC) or extraintestinal pathogenic *E. coli* (ExPEC). The IPECs are major diarrhoeagenic pathogens that cause gastroenteritis with six intestinal pathotype subgroups: enterohemorrhagic *E. coli* (EHEC), enteropathogenic *E. coli* (EPEC), enteroaggregative *E. coli* (EAEC), enterotoxigenic *E. coli* (ETEC), enteroinvasive *E. coli* (EIEC), and diffusely adherent *E. coli* (DAEC). Meanwhile, ExPEC consists of three human pathotype subgroups: neonatal-meningitis *E. coli* (NMEC), uropathogenic *E. coli* (UPEC), sepsis-associated pathogenic *E. coli* (SePEC), and the avian pathogenic *E. coli* (APEC) (4).

Studies have shown that *E. coli* is a highly adaptable bacterium that can survive and grow in hostile external environments-niches (5, 6). Environmental *E. coli*, from aquatic riverine environments, are genetically diverse (7) and with the current application of a PCR-based diagnostic method, seven major phylogenetic groups (A, B1, B2, C, D, E, and F) and five cryptic clades are categorized (8, 9). These different phylogroups have different associations with phenotypic and genotypic traits, metabolic properties, ecotype, lifestyle, and pathogenicity (10–13).

Studies have found that antibiotic-resistant (AR) *E. coli* are ubiquitous in the aquatic environment and the rising emergence of multiple AR (MAR) *E. coli* (14, 15), coupled with the potential presence of virulent strains, is a great concern for public health (16). The antibiotic residues, AR *E. coli*, and their resistome concomitantly enter into the aquatic environment through wastewater discharge from anthropogenic activities (17, 18). Thus, aquatic ecosystems may serve as reservoirs that provide an ideal setting for the acquisition and dissemination of AR resistome between the environmental bacterial communities and non-pathogenic and pathogenic bacteria *via* horizontal gene transfer (19–21), which is also known as a lateral gene transfer (22).

Despite the different studies on *E. coli* from different aquatic ecosystems, phenotypic and genotypic studies of antibiotic resistance of these bacteria, and their distribution remain scarce, particularly in tropical aquatic ecosystems. The rivers of Asia are amongst the most polluted in the world and contain up to three times as much bacteria from human waste, where the reported fecal count is 50 times that of the WHO guidelines (23). In Malaysia, the major causes of pollution in rivers are related to anthropogenic activities and the sources of contamination are mainly attributed to industrial areas, sewages, workshops, residential areas, animal, and agricultural farming activities according to the Department of Environment (DOE), Malaysia (24, 25). These anthropogenic influences subsequently lead to the deterioration of water quality with elevated concentrations of heavy metals, mercury, coliforms, and nutrient loads (26).

Ghaderpour et al. (27) reported the prevalence of diverse AR *E. coli* in the Larut River estuarine waters, with one of the largest mangrove forests in Malaysia, and suggested anthropogenic sources as the major contributor of antibiotic resistance. Larut River is the only river flowing through the town of Taiping, Perak. This study aimed to determine the impact of anthropogenic wastewaters from a hospital, a zoo, and a poultry slaughterhouse in the town of Taiping to the occurrence, genetic diversity, and virulence of AR *E. coli*, as well as their resistance genes in the riverine estuarine waters of Larut River.

## Materials and Methods

### Sampling Sites

**Figure 1:**
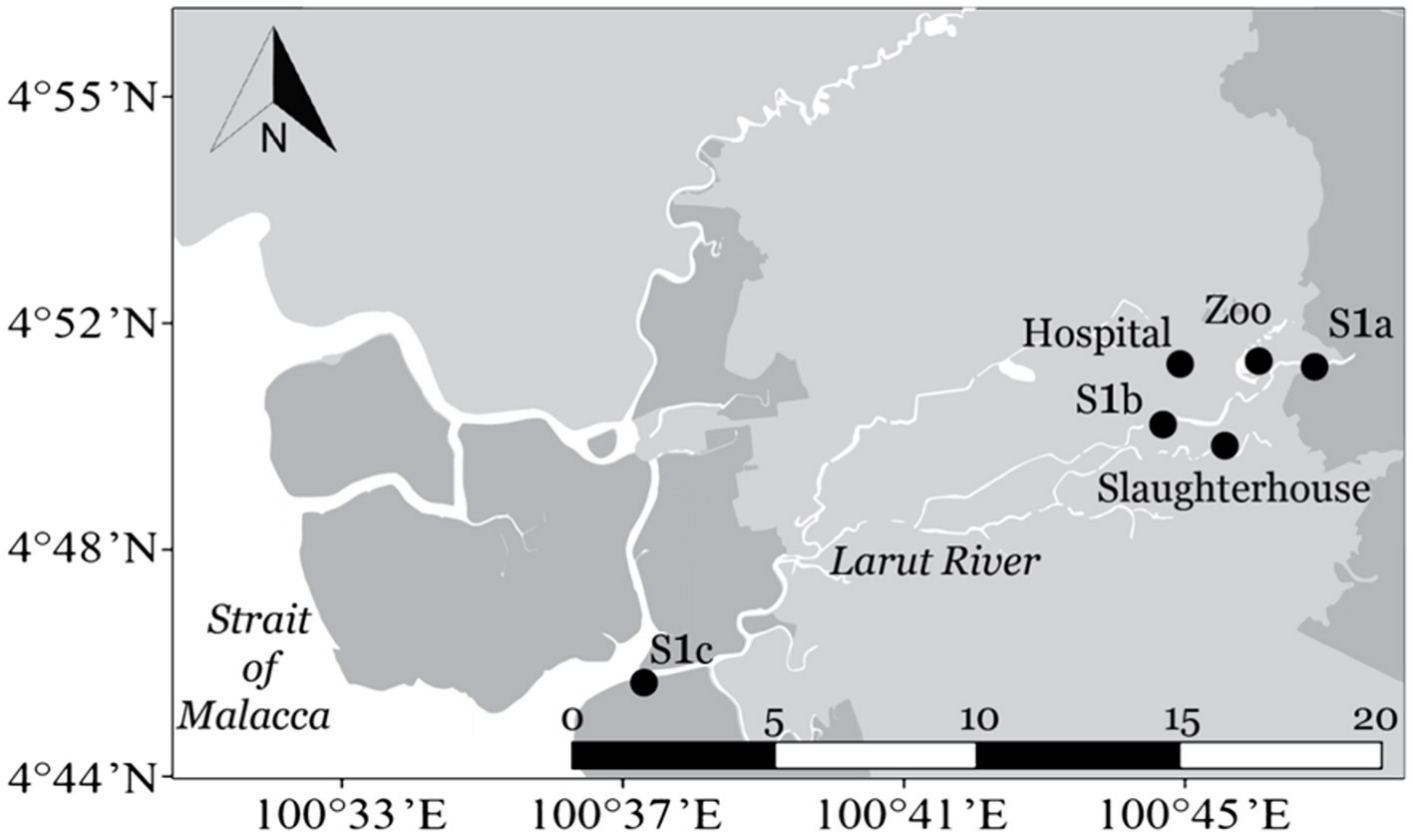
Map of sampling sites along Larut River. Geolocation: S1a (4°51.158'N, 100°45.737'E), Zoo (4°51.101'N, 100°45.045'E), Hospital (4°51.149'N, 100°44.018'E), Slaughterhouse (4°50.238'N, 100°44.709'E), S1b (4°50.535'N, 100°43.925'E), and S1c (4°50.140'N, 100°37.583'E).

Sampling was conducted at six sampling sites located in the upstream, middle, and downstream of the Larut River. Larut River is 20.9 km long, serving a population of approximately 217,647 (28). Water samples were collected from upstream (S1a, 04°51.158'N, 100°45.737'E), at the reserve forest Larut Hill (elevation: 1,250 m) followed by the middle downstream where the river water received wastewater discharges from a zoo (04°51.101'N, 100°45.045'E), a public hospital (04°51.149'N, 100°44.018'E), and a slaughterhouse (04°50.238'N, 100°44.709'E) before passing through downstream Larut (S1b, 04°50.535'N, 100°43.925'E) and finally reaching the Larut Estuary (S1c, 04°50.140'N, 100°37.583'E) (Figure 1).

### Sample Collection and Physicochemical Analysis

The greywater effluents, with possible fecal contamination, were collected at the main outlet of a hospital, a slaughterhouse, and a zoo while the river surface waters were collected from S1a, S1b, and S1c of the Larut River. The water samples were collected in triplicates from each sampling site using sterilized 2 L amber glass bottles and were kept on ice until further analysis in the laboratory. A simultaneous study was conducted to measure the *in situ* physicochemical parameters [temperature, salinity, pH, and dissolved oxygen (DO)] and the dissolved inorganic nutrients [nitrate (NO_3_), nitrite (NO_2_), ammonium (NH_4_), phosphate (PO_4_), and silicate (SiO_4_)]. The physicochemical results were published earlier in Lye et al. (29).

### Isolation and Enumeration of Coliform and *E. coli*

For the isolation and enumeration of coliform and *E. coli*, the membrane filtration technique with the CHROMagar^TM^ ECC media (CHROMagar Inc., Paris, France) was used. After filtering a suitable volume of sample onto a pre-sterilized 0.45 μm nitrocellulose membrane, the membrane filter was transferred onto the CHROMagar^TM^ ECC media and incubated at 37 ± 0.5°C overnight. Blue colonies were enumerated as *E. coli*, whereas mauve colonies were enumerated as total coliform. Presumptive *E. coli* isolates were further purified with Luria Bertani medium and were preserved in stab and glycerol solution before further tests. The abundance of total coliform and *E. coli* were reported as colony-forming units per 100 ml (CFU 100 ml^−1^).

### *E. coli* Identification and Detection of Virulence Genes

The identity of presumptive *E. coli* isolates was confirmed by a monoplex PCR targeting the *E. coli* housekeeping gene [alkaline phosphatase (*phoA*) gene] using primers and amplification conditions as described by Kong et al. (30). Detection of virulence genes was performed by two multiplex PCR assays, in which nine virulent genes were for the differentiation of six *E. coli* pathotypes. The two multiplex PCR was conducted in a 25 μl of reaction mixture consisting of 5 μl DNA, 1× Green GoTaq buffer (pH 8.5), 0.5 U of Taq DNA polymerase (Promega, USA), 1.65 mM of MgCl_2_, 220 μM dNTP, and 0.24 μM of each primer. The primers used and the amplification conditions were adopted from Nguyen et al. (31), Vidal et al. (32), and Aranda et al. (33) (Supplementary Table 1). The *E. coli* 2060-004, E2348/69, JM221, E9034A, C1845, and EC-12 were used as positive controls for EHEC, EPEC, EAEC, ETEC, DAEC, and EIEC, respectively, whereas sterile water was used as the negative control.

### Phylogenetic Grouping of *E. coli*

A quadruplex PCR assay was performed to determine the phylogroups (A, B1, B2, C, D, E, and F) that belong to *E*. *coli sensu stricto*, or one of the five cryptic clades (I-V) using primer concentrations and PCR conditions according to Clermont et al. (9) (Supplementary Table 1). For each quadruplex reaction, 20 μl reaction volume was carried out containing 3 μl DNA template, 2 μM each dNTP, 1X PCR buffer, 2 U Taq polymerase, 1 mM MgCl_2_, 1 μM for primers except for Acek-F (2 μM), ArpA1-R (2 μM), trpBA-F (0.6 μM), and trpBA-R (0.6 μM). The combination of presence or absence of the genes *arpA, chuA, yjaA*, and TspE4.C2 was used to determine the phylogenetic groups according to Clermont et al. (9).

### Antimicrobial Susceptibility Tests

The disc diffusion method on Mueller-Hinton agar (Difco, USA), as described by the Clinical Laboratory Standards Institute (34), was used to determine the antibiotic resistance profiles of the *E. coli* isolates. A total of 20 commonly used antibiotics from 11 classes were tested: tetracyclines [tetracycline (TE) 30 μg], quinolones [nalidixic acid (NA) 30 μg, oxolinic acid (OA) 2 μg, and ofloxacin (OFX) 5 μg], penicillins [ampicillin (AMP) 10 μg, amoxicillin/clavulanic acid (AMC) 30 μg], sulfonamides [sulfafurazole (SF) 300 μg and sulfamethoxazole/trimethoprim (SXT) 25 μg], fluoroquinolones [ciprofloxacin (CIP) 5 μg, ofloxacin 5 μg, and enrofloxacin (ENR) 5 μg], phenicols [chloramphenicol (C) 30 μg and florfenicol (FFC) 30 μg], aminoglycoside [streptomycin (S) 10 μg, gentamicin (CN) 10 μg, and neomycin (N) 30 μg], macrolide [azithromycin (AZM) 15 μg], cephalosporins [cephazolin (KZ) 30 μg and ceftriaxone (CRO) 30 μg], carbapenems [imipenem (IPM) 10 μg], and nitrofurans [nitrofurantoin (F) 100 μg]. The antibiotic discs were applied to the inoculated plates and were incubated at 37 ± 0.5°C overnight. The zone of inhibition for each *E. coli* isolate was analyzed according to the standards and the interpretive criteria of CLSI (34). The *E. coli* ATCC 25922, which is a recommended reference strain for antimicrobial susceptibility testing, was used as a control.

### Multiple Antibiotic Resistance Index

The average MAR index of all *E. coli* isolates was calculated using the following formula:


(1) $$\begin{array}{l} \text{MARindex}=\text{ a}/\text{bc} \end{array}$$

where “a” is the aggregate antimicrobial resistance score of all the *E. coli* isolates from one site, “b” is the number of *E. coli* isolates tested, and “c” is the number of antibiotics used in the study. A MAR index value of ≥0.2 indicates that the antibiotic resistance at the study area is rendered from contamination by antibiotics, whereas the value of <0.2 indicates that the antibiotic resistance at the study area is indigenous (35).

### Antibiotic Resistance Gene Detection

In this study, we assessed tetracycline and sulfonamide resistance genes as both tetracycline and sulfonamide are old antibiotics that were among the most widely used antibiotics in humans and animals particularly in Southeast Asia, either as therapeutic and/or prophylactic agents (36). For *tet* gene detection, multiplex PCR was used to screen 14 *tet* genes (37). All reaction mixtures were performed in 50 μl volume consisting of 2 μl DNA template, 1× PCR buffer, 2.5 U Taq polymerase, 300 μM of each dNTPs, 3 mM MgCl_2_, and selected primers with 0.2 μM for *tet*(*D*), 0.4 μM for *tet*(*M*) and *tet*(*S*), 0.6 μM for *tet*(*A*), *tet*(*B*), *tet*(*E*), *tet*(*G*), *tet*(*K*), *tet*(*O*), 0.8 μM for *tet*(*C*), *tet*(*L*), *tet*(*Q*), *tet*(*X*), and 1.6 μM for *tetA*(*P*). While for *sul* genes, duplex and monoplex PCR were performed according to Kozak et al. (38) and Pei et al. (39) by using gene-specific primers of *sul1, sul2*, and *sul3*, respectively. The PCR amplifications were carried out in 25 μl volume containing 1× PCR buffer, 1 mM MgCl_2_, 0.2 μM each primer, 2 μl DNA template, 1.5 μM of each dNTPs, and 2 U Taq polymerase for *sul1* and *sul2;* while the reaction mixture for *sul3* contained 1× PCR buffer, 1 mM MgCl_2_, 0.2 μM each primer, 1 μL DNA template, 0.2 μM of each dNTPs, and 1.75 U Taq polymerase.

### Genetic Diversity Determination *via* REP-PCR

The genetic diversity of the *E. coli* isolates was analyzed by a Repetitive Extragenic Palindromic-PCR (REP-PCR) using REP oligonucleotides as previously reported by Lim et al. (40). The *E. coli* isolates were prepared, and the PCR amplification was essentially conducted as described by Ghaderpour et al. (27). Amplifications were carried out in 25 μl volume including 4 μL DNA template, 200 μM of each dNTPs, 1× PCR buffer, 1 U Taq polymerase 2.5 mM MgCl_2_, and 0.5 μM primer. The fingerprint patterns were analyzed by BioNumerics, version 7.6.3 (Applied Maths, Kortrijk, Belgium). The similarity between profiles was calculated with the dice coefficient, while cluster analysis was performed using the unweighted pair group method using arithmetic averages (UPGMA) with Shannon diversity index (H'). Shannon diversity index (H') was calculated using the following equation:


(2) $$H^{\prime }=-\sum \;Pi\text{ log }Pi$$

(3) Pi=ni/N

where *ni* is the number of strains having each band pattern, N is the total number of isolates applied for rep-PCR.

### Statistical Analysis

Statistical analysis was performed with the SPSS version 21.0 (IBM, Chicago, USA). The criterion for statistical significance for all the following analyses was at *p* ≤ 0.05. Pearson's chi-square (goodness of fit) test was performed to determine the significant difference among phylogenetic groups, *sul* resistance genes, and *tet* resistance mechanism types according to frequency data, whereas the chi-square test for independence was applied to determine any association between phylogroups and antibiotic resistance. Prevalence of antibiotic resistance was defined as the proportion of resistant *E. coli* isolates over the total tested isolates. Correlation and linear regression analyses were performed to establish any association between water quality (29) toward *E. coli* and total coliform abundance. Cluster analysis for sampling sites was performed based on the antimicrobial susceptibility profile through the Bray-Curtis similarity index using PAST version 3.22 (41). Besides that, canonical correlation analysis (CCA) was performed using PAST to analyze the distribution of *E. coli* phylogenetic groups among sampling sites relative to the resistance genes *sul* and *tet*, water quality parameters, and antibiotic residue concentrations (42).

## Results and Discussion

### Abundance of Coliform and *E. coli*

**Figure 2:**
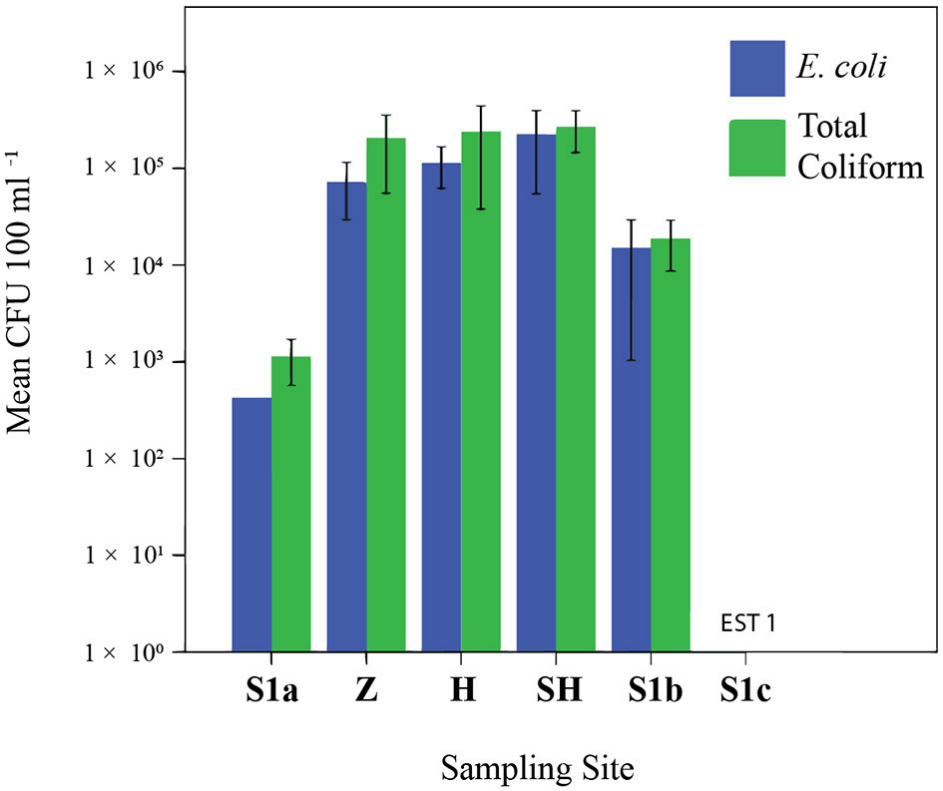
Abundance of *Escherichia coli* and total coliform. EST, estimation. Sampling sites: Z, Zoo; H, Hospital; SH, Slaughterhouse.

Coliform and *E. coli* were detected at all sampling sites; their concentrations at Larut River are up to 4.7 × 10^5^ CFU 100 ml^−1^; up to 4.1 × 10^5^ CFU 100 ml^−1^, respectively (Figure 2). The results clearly showed that Larut River was affected by anthropogenic wastewaters that harbored more coliform and *E. coli* (1–5 log CFU 100 ml^−1^ difference) compared to river waters (*p* ≤ 0.05). The highest total coliform and *E. coli* counts were observed in wastewater effluent from the slaughterhouse. In addition to lower anthropogenic influence, the comparatively low counts of coliform and *E. coli* detected at S1c were associated with the inhibitory effect of salinity (>16 ppt) on their survival and growth rates (29, 43), as coliform counts decreased significantly with increasing salinity in Larut River (*R*^2^ = 0.26, df = 16, and *n* = 18). The coliform and *E. coli* concentrations detected in this study were within the range that is previously reported for polluted Malaysian rivers and other locations (18, 44–47), which exceeded the standard maximum 100 CFU 100 ml^−1^ limit in the National Water Quality Standards (NWQS) class II for rivers set by the DOE Malaysia and the Malaysia Interim Marine Water Quality Standards (25). The important sources of fecal pollution in the Larut River are poultry manure, agriculture runoff, poorly treated or untreated sewage, and anthropogenic input from human and industrial activities along the river basins that contributed to the deterioration of water quality.

### Distribution of *E. coli* Phylogenetic Groups

A total of 354 *E. coli* were isolated, and all the seven phylogenetic groups were observed with no cryptic clades. The majority of the *E. coli* isolated (77.97%) from Larut River were commensal strains, with phylogroup B1 (39.55%) and phylogroup A (38.42%) among the most prevalent. The predominance of groups A and B1 were consistent with other studies (27, 48). These two groups are sister groups that are consistently more predominant and prevalent in aquatic environments than virulent extraintestinal strains from groups B2 and D (27, 48). The phylogroup B1 was found higher at the wastewaters from zoo, slaughterhouse, and S1c of the Larut River, thus, receiving anthropogenic discharges from upstream. This may be associated with the influence of effluents discharge that contained more animal waste as the phylogroup B1 tend to be isolated from animal feces, particularly from herbivorous animals (49). Furthermore, this group of bacteria has already been shown to benefit from longer persistence in water than other phylogroups albeit for estuarine and coastal waters (6, 44, 50). In this study, it was expected to find a higher frequency of human-associated phylogroup A in wastewater effluent from the hospital, followed by slaughterhouse and S1b, which received the wastewater from the same point source. The least abundant and rarely detected phylogroups B2, C, D, E, and F isolated from Larut River were consistent with other studies (51, 52). The phylogroups B2 and D are known to be related to virulence factors that cause extraintestinal infections. Their higher detection frequencies at upstream (S1a) (B2: χ^2^ = 74.799, df = 5, *p* = 0.000; D: χ^2^ = 11.094, df = 5, and *p* = 0.050) probably resulted from commensal isolates from birds (53) and wild mammals (54, 55), as Larut Hill Forest Reserve is rich in biodiversity as it shelters 227 bird and 27 mammal species (56). This is in agreement with Petit et al. (57) and Ghaderpour et al. (27) who reported a higher abundance of B2 and D phylogroup at surface water collected near a forest with lesser human activities. Thus, fecal pollution from wildlife could present pathogenic *E. coli* infection risks. Hence determining the sources of fecal pollution in environmental waters is essential for pollution control and sustainable water quality management. The distribution of phylogenetic groups was not homogenous among the sites, and this may be attributed to the land use, sources of pollution, selective pressures in the waters, availability of nutrients, *in situ* physicochemical parameters (dissolved oxygen, pH, salinity, etc), protozoan and bacterial predators, and hydrological conditions (46, 58). The different survival abilities of *E. coli* and their abilities to overcome stresses will structure their community distribution and diversity in aquatic environments (6).

### Antimicrobial Susceptibility Profile

**Figure 3:**
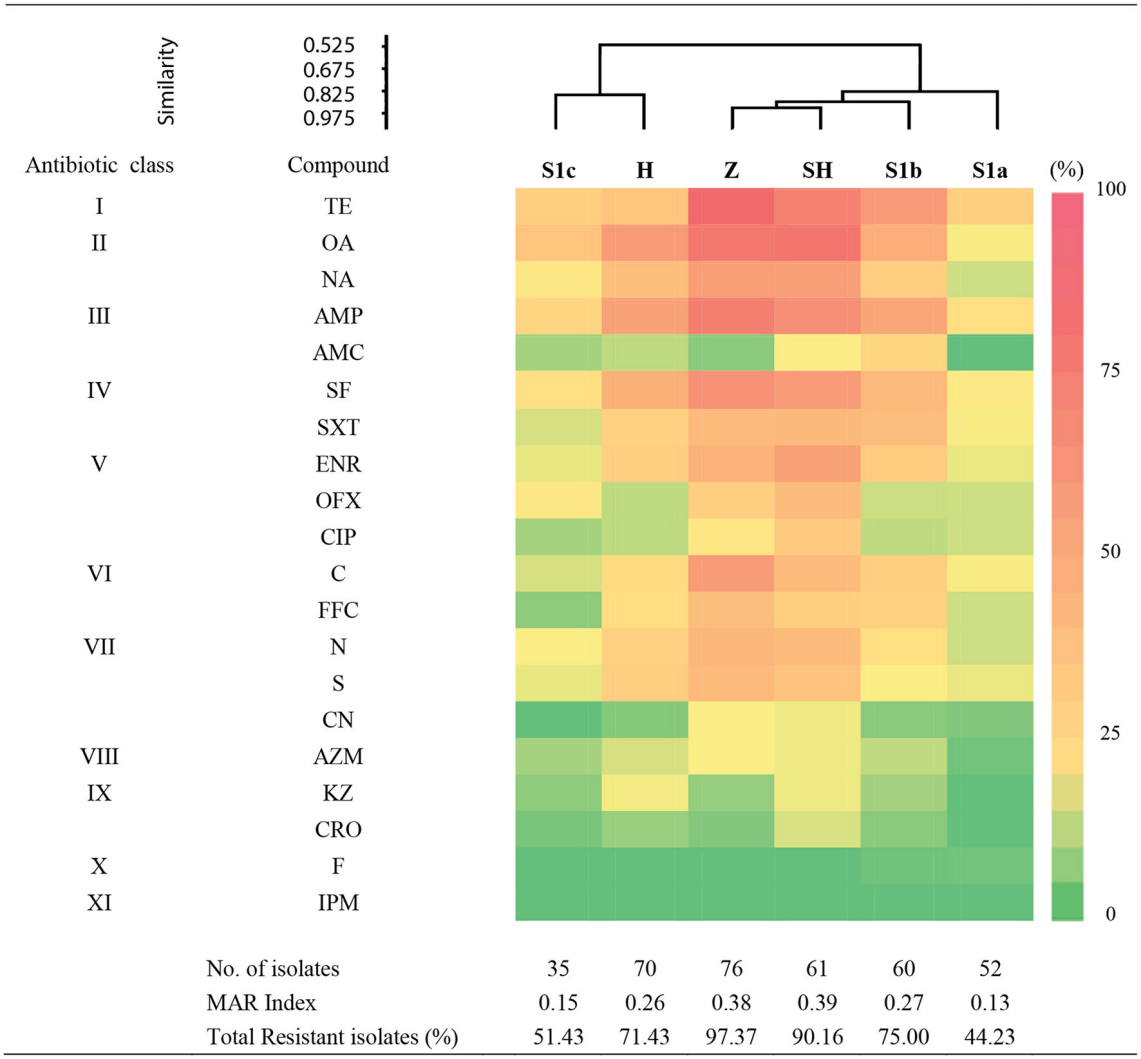
Antibiogram of antibiotic resistance phenotype detected in surface water among sampling sites in Larut River organied by similarity. Abbr, abbreviation. Antibiotic class: I, Tetracycline; II, Quinolone; III, Penicilin; IV, Sulfonamide; V, Fluoroquinolone; VI, Amphenicol; VI, Aminoglycoside; VIII, Macrolide; XI, Cephalosporin; X, Nitrofuran; XI, Carbapenem. Antibiotic type: TE, Tetracycline; OA, Oxolinic acid; NA, Nalidixic acid; AMP, Ampicilin; AMC, Amoxycillin/Clavulanic acid; SF, Sulfafurazole; SXT, Sulfamethoxazole/trimethoprim; ENR, Enrofloxacin; OFX, Ofloxacin; CIP, Ciprofloxacin; C, Chloramphenicol; FFC, Florfenicol; N, Neomycin; S, Streptomycin; CN, Gentamicin; AZM; Azithromycin; KZ, Cephazolin; CRO, Ceftriaxone; F, Nitrofurantion; IPM, Imipenem. Sampling sites: Z, Zoo; H, Hospital; SH, Slaughterhouse.

We examined the susceptibility of all the isolated *E. coli* in this study with 20 antibiotics that represented 11 different antibiotic classes. Of them, the highest frequency of resistance was detected for tetracycline class (34.32–86.84%), followed by quinolones (13.46–80.33%), penicillins (0–75%), sulfonamides (14.29–65.79%), amphenicols (5.71–60.53%), fluoroquinolones (8.57–57.38%), and aminoglycosides (0–47.37%) (Figure 3). The resistance rates against macrolides (1.92–19.74%), cephalosporins (0–18.57%), and nitrofurans (0–1.92%) were low. All the isolates were susceptible to imipenem and none of the isolates were resistant to all the antibiotics tested. Among these isolates, 265 (74.86%) were confirmed resistant to at least one or more antibiotics, two MAR *E. coli* isolates from phylogroups F and B1, each isolated from the slaughterhouse and hospital effluents, respectively, were resistant against 16 types of antibiotics. Only 25.14% of the isolates were susceptible to all antibiotics tested, indicating the prevalence of AR *E. coli* in the Larut River. Our results are consistent with other studies that reported the resistance of *E. coli* against old and widely used antibiotics in aquatic environments (27, 59–61). Furthermore, these antibiotic classes, except aminoglycosides, were the frequently detected antibiotics in Larut River (antibiotic concentration: LOD−1,092.49 ng/L) (29, 42). Our results showed that the frequency of MAR was higher in wastewater effluents (zoo > slaughterhouse > hospital) than in river waters (S1b > S1c > S1a). The prevalence of environmental MAR *E. coli* detected in this study was comparable to Kat River in South Africa (62) and Cochin Estuary in India (63), but higher than Matang Estuary in Malaysia (27), Tagus Estuary in Spain (18), Kshipra River in India (64), San Pedro River in Mexico (65), and generally lower than DongJiang River in China (88.00%) (66).

Cluster analysis revealed that the distribution of antibiotic resistance phenotype of *E. coli* isolates from the zoo and the slaughterhouse effluents were more similar than the hospital effluent and S1c. This could be due to the influence of wastewater effluent discharge that contained animal waste that received similar and commonly used veterinary antibiotics as previously reported by Low et al. (42). In contrast, the almost similar distribution of phylogenetic groups, together with the wastewater effluents containing human waste, could be a contributing factor to the similarity of antibiotic resistance phenotypes from the zoo and slaughterhouse effluent compared to S1b, which received the anthropogenic inputs from upstream. In this study, the phylogroup with the most resistant strains was observed in B1 followed by A, B2, D, E, F, and C. Our findings were consistent with other studies that MAR of *E. coli* mostly belonged to phylogroups B1 and A (27, 67). Despite these groups generally being considered harmless, their prevalence as intestinal pathogenic strains (68) has increased the risk on public health. The lower percentage of antibiotic resistance in phylogenetic groups B2 and D were also consistent with Ghaderpour et al. (27). These phylogroups were detected more upstream (S1a), which was associated with an animal origin. Phylogroup B2 was found to have higher resistance against penicillin, quinolones/fluoroquinolones, sulphonamides, and aminoglycosides, whereas Phylogroup D was detected to have higher resistance against tetracycline, penicillin, and sulfonamides. Studies have revealed their occurrence in wild, companion, and food animals, suggesting diet may be the main factor introducing MAR bacteria to animals (55, 69–72). Besides, rapid urbanization causes habitat loss and has simultaneously exposed animals to various environmental pollutants, which subsequently favors the transfer and emergence of MAR bacteria in animals through the food chain (72–74). The MAR index of 0.2 was observed at all the sampling sites, except for S1a and S1c, indicating that the midstream Larut River is contaminated with different anthropogenic inputs from the hospital, zoo, and slaughterhouse effluents that contained high risks sources, which were able to influence the prevalence and phylotype diversity of MDR *E. coli* in the Larut River.

### Pathotypes of *E. coli*

Recent studies began using virulence factors as risk indicators in the environment, due to their close relation to pathogenicity among *E. coli* isolates (75). We found that 9 environmental *E. coli* isolates harbored an intestinal pathogenic *E. coli* (IPEC)-associated virulence gene. The relatively low abundance of presumptive IPEC in this study concurred with other aquatic environments with non-point source contamination (27, 44, 76), but contrasted with waters impacted by a sewage treatment plant (77). The virulence factor *aggR*, which indicates a positive EAEC pathotype, was the most prevalent gene (*n* = 5) detected in effluents from the zoo and the hospital. Runoff from sewage overflows likely transported these *E. coli* isolates, with virulent factors from feces of animals and humans, into environmental waters. Additionally, *bfpA* (*n* = 1), ST (*n* = 2), and *eae* (*n* = 1) virulence genes were also detected along the river continuum with high susceptibility to antibiotics except for a single phylogroup F isolate from zoo effluent. Nevertheless, these detected virulence genes still play their respective roles in lesion attachment, adherence, ion outflow induction, etc. (78). Although these *E. coli* isolates each carried a virulence gene, the observation of virulence factors itself does not always demonstrate pathogenicity (79). The present detection of single virulence gene patterns in *E. coli* isolates could likely be explained by a horizontal gene transfer among cells, which mediate the exchange of virulence factors located on mobile genetic elements (80). We identified five *E. coli* isolates (2.19%) from the zoo (*n* = 3) and hospital (*n* = 2) effluents to be EAEC strains. These are emerging diarrheal pathogens that cause acute diarrhea in developing countries (81). These EAEC isolates possessed MAR profiles of 5–16 antibiotics, with combinations of *sul2* and *sul3* genes, and *tet*(*B*), *tet*(*A*), *tet*(*L*), *tet*(*M*), and *tet*(*X*) genes. Studies have revealed similar findings on EAEC strains to be associated with water surfaces (82) and MAR EAEC belonging to phylogroup F from a stream with poor water quality (83). Our finding suggested that the dissemination of both virulence and resistance determinants could occur in the same anthropogenic site and pose a health risk. Either resistant or pathogenic isolates, when in contact with autochthonous bacteria, may then disseminate resistance and virulence determinants among natural ecosystems *via* a horizontal gene transfer (84). Interestingly, the phylogroup F strains isolated (*n* = 4/9) were found to carry virulent genes. El-shaer et al. (85) found that although environmental isolates which harbored virulence genes were located in phylogroup B1, the newly described phylogroup, such as phylogroup F, has also a virulence potential. The impact of this observation is not well-elucidated and further study is needed. Members of these phylogenetic groups are of particular interest because there is a relationship between the genetic background of a strain and its virulence factors (86).

### *tet* and *sul* Gene Distribution in *E. coli*

**Figure 4:**
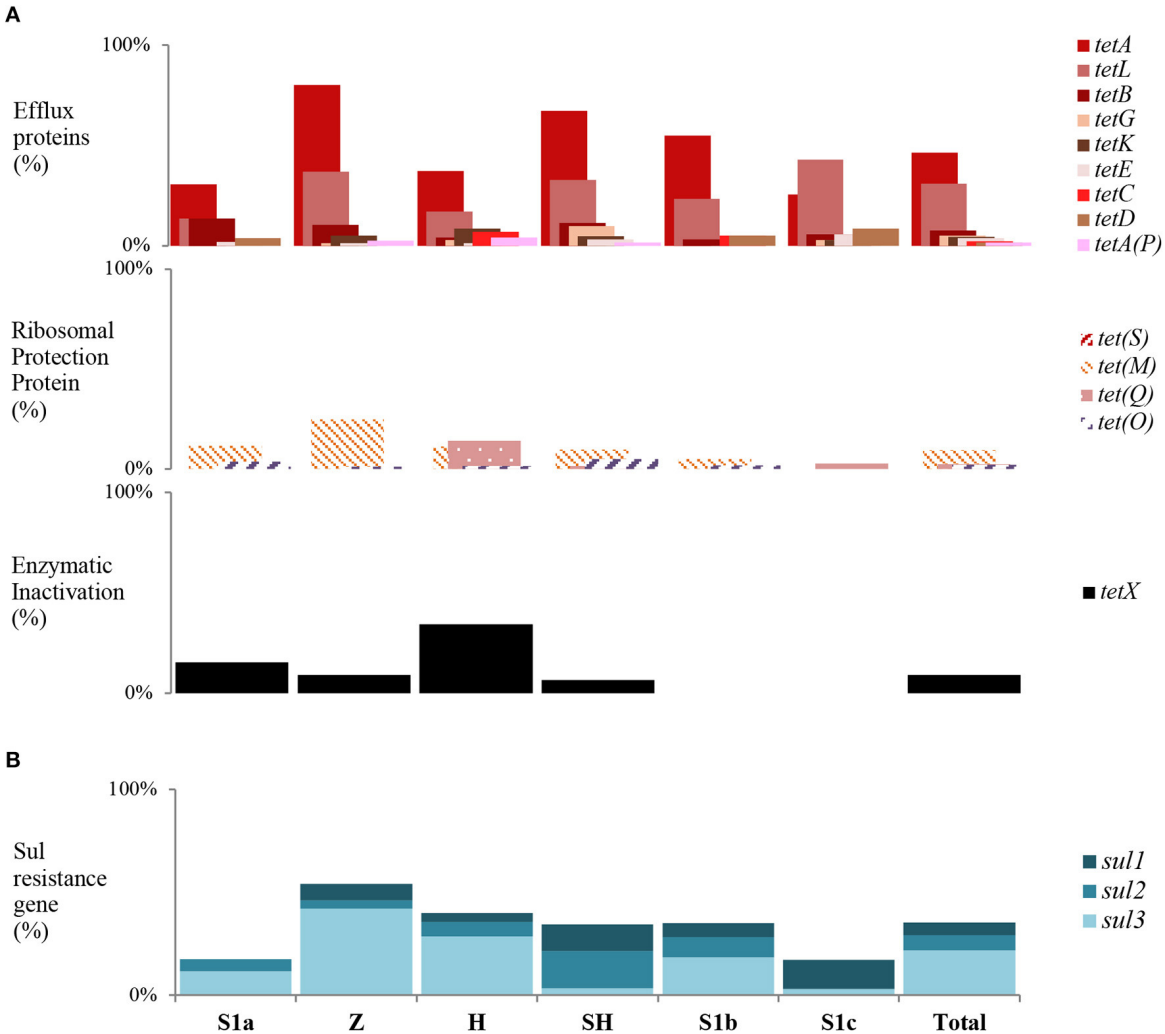
Trends of *tet* and *sul* gene among sampling sites in Larut River. **(A)** *tet* resistance gene type detected by site **(B)** Cumulative *sul* resistance gene type detected by site. Z, Zoo; H, Hospital; SH, Slaughterhouse.

Overall, 277 (78.25%) *E. coli* isolates harbored at least one of the tested *tet* genes except for *tet(S)*. The majority of the resistance genes were found in the zoo (93.42%) effluents, slaughterhouse (85.25%) effluents, and S1b (78.33%). Seventy-seven *E. coli* isolates did not harbor any of the tested resistance genes. The three predominant *tet* genes were *tet*(*A*) (52.64%), followed by *tet*(*L*) (27.12%), and *tet*(*X*) (12.15%) (Figure 4A). Among sites, *tet*(*A*) was the dominant gene in zoo effluent, *tet*(*L*) was abundant in S1c, and *tet*(*X*) was in hospital effluent. We found that efflux genes were prevalent among the sites indicating that the active efflux *via* membrane-associated proteins were the main mechanisms for resistance in *E. coli* that resided in the Larut River. Both *tet*(*A*) and *tet*(*L*) belonged to the active efflux resistant mechanism. The predominance of these genes could be due to the low concentrations of tetracycline [mean: 64.4 ng L^−1^ (42)] in the environment as the expression of these genes are mainly induced at low tetracycline level (87). Both resistance mechanisms of active efflux *via* membrane-associated proteins, along with ribosomal protection proteins (RPP), were found in zoo wastewater effluent. The expression of the RPP *tet*(*M*) is mainly induced only at the high tetracycline level (88). This fact could explain the overall low abundance of *tet*(*M*) detected in this study. Certain *E. coli* (37.85%) in this study were found to carry multiple *tet* resistance genes with a high variation of *tet* gene combination. Our findings concurred with previous studies that tetracycline-resistant genes are ubiquitous in aquatic environments (89, 90). Among the 57 different *tet* combinations harbored by *E. coli* isolates, the most prevalent multiple *tet* resistance genes [*tet*(*A*)(*L*) (8.19%), *tet*(*A*)(*M*) (5.65%), and *tet*(*A*)(*L*)(*M*) (4.24%)] were significantly dominant (*p* ≤ 0.05) in wastewater effluents from the hospital. Notably, we had detected the presence of all three *tet* resistance mechanisms that include enzyme inactivation [tet(*X*)] in *E. coli* isolates isolated from hospital effluent. In contrast, *E. coli* isolates that harbored significantly fewer resistance genes were observed at less polluted sites. Our results indicated that tetracycline-resistant *E. coli* acquired multiple mechanisms to confer resistance.

For the sulfonamide resistance gene, there were 125 (35.31%) *E. coli* isolates that harbored at least one among all sites, with the gene-frequency distribution of *sul3* (20.90%) > *sul2* (5.08%) > *sul1* (3.11%) (Figure 4B). Among the multiple *sul* gene combinations, the *sul1* and *sul3* combination was the most frequently detected in this study. The pattern of gene frequency distribution observed concurred with Lai et al. (91), where *sul3* was prevalent in urban freshwater aquatic recipients of Sweden. The *sul1* gene has been widely documented in receiving aquatic environments that can be regarded as a marker for anthropogenic pollutants (92, 93). Meanwhile, *sul3* is initially suspected to be of human origin (94), but numerous studies have reported their prevalence in *E. coli* isolates from animals and livestock (95–97). Thus, the widespread of *sul3* among *E. coli* isolates in this study was most likely due to the consumption of sulfonamide by humans and veterinary use (29, 36, 42). Sulfonamides are widely used in Asian countries, and in Malaysia, the usage in veterinary medicine was ~18,000 kg per year (98). While for the healthcare industry, the usage ranged from 0.0982 to 5.9900 daily defined dose (DDD)/1,000 population/day (99). In the absence of selective pressure from sulfonamide, sulfonamide-resistant bacteria may remain stable in the environment for at least 5–10 years longer than sulfonamide itself (100). The sulfonamide-resistant phenotypes that carried no resistant determinants was most likely due to the acquisition of other mechanisms (e.g., mutations in the chromosomal DHPS gene *flop*), as environmental *E. coli* strains may acquire genetically unrecognized resistance mechanisms more frequently compared to clinical *E. coli* strains (101, 102).

**Figure 5:**
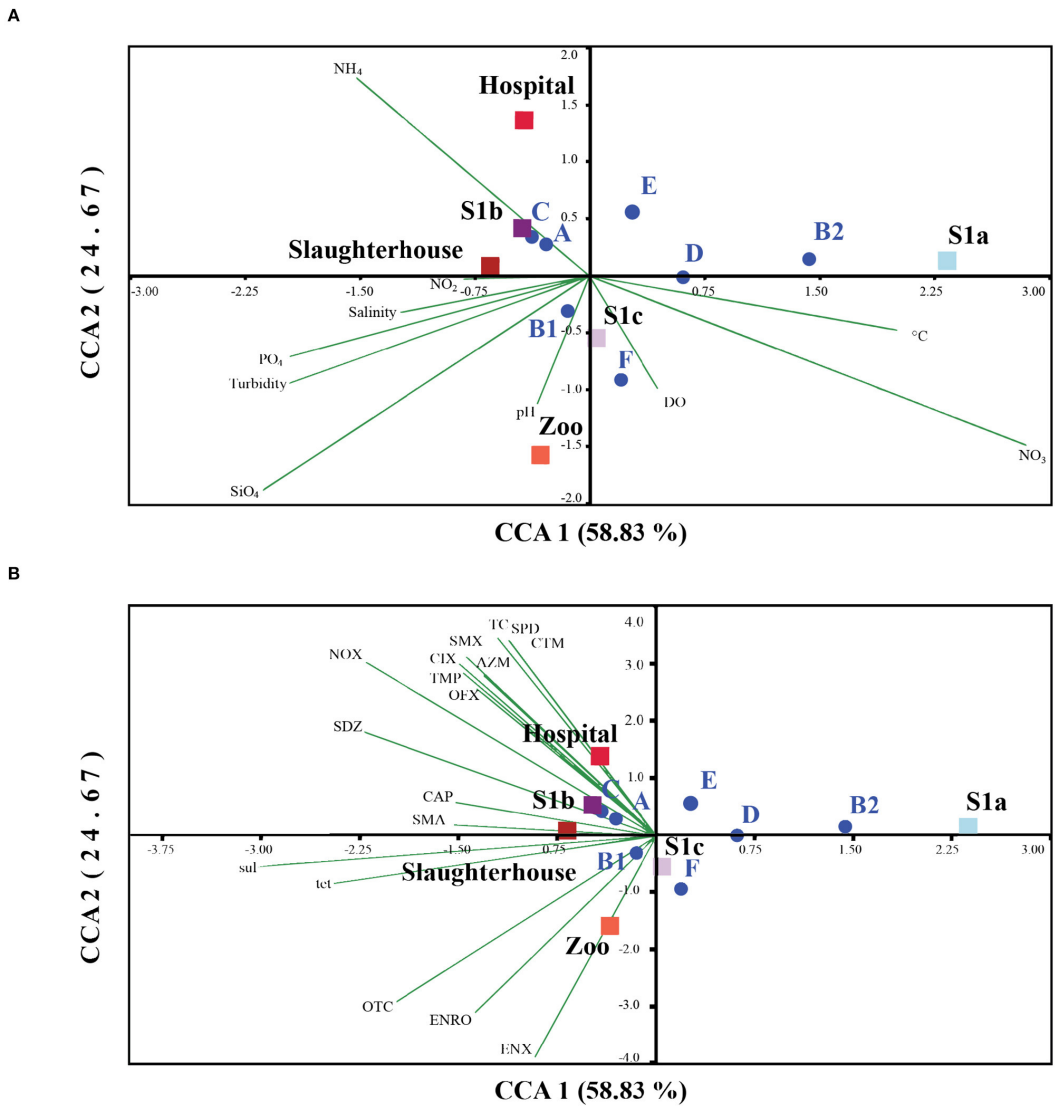
Square root normalized CCA ordination biplot showing the distribution of *E. coli* phylogenetic groups by sampling sites in Larut River with respect to their phylogenetic group and resistance genes *tet* and *sul*. CCA 1 on axis 1 explained 52.60% whereas CCA 2 on axis 2 explained 25.81% of all explanatory variables. **(A)** Relationship between *E. coli* and water physicochemical parameters of Larut River collected by Lye et al. (29). **(B)** Relationship between *E. coli* and water antibiotic residue detected in Larut River collected by Low et al. (42). Water physicochemical factors abbreviation: Temp (°C), salinity (ppt), pH, dissolved Oxygen (DO), turbidity and concentrations (μM) of Silicate (SiO_4_), Ammonium (NH4), Nitrite (NO_2_), Nitrate (NO_3_), Phosphate (PO4). Antibiotic resistance gene: *sul*, sulfonamide resistance gene; *tet*, tetracycline resistance gene. Antibiotic residue: RTM, Roxithromycin; CTM, clarithromycin, AZM, azithromycin; ETM-H_2_O, erythromycin-H_2_O; ENX, enoxacin; ENRO, enrofloxacin; NOX, norfloxacin; OFX, ofloxacin, CIX, ciprofloxacin; CTC, chlortetracycline; OTC, oxytetracycline; TC, tetracycline; FF, florfenicol; CAP, chloramphenicol; TMP, trimethoprim; SAAM, sulfacetamide; STZ, sulfathiazole; SDM, sulfadimethoxine; SMA, sulfadimidine; SPD, sulfapyridine; SDZ, sulfadiazine; SMX, sulfamethoxazole.

Relationship between physicochemical and antibiotic residues on *E. coli* phylogenetic distribution studies have shown that *E. coli* phylogenetic groups are adaptable and genotypically affected by environmental changes (103). In our study, the correlation coefficient has shown that salinity explained 26% of the variation in coliform abundance. Thus, other factors in combination may also contribute to the distribution of *E. coli* and their activity in the environment. Our CCA analysis revealed that the *E. coli* phylogroups A, B1, and C were greater in deteriorating water quality with SiO_4_, NH_4_, and PO_4_ (Figure 5A). The phylogroup B1 seemed to grow well in turbid water, with NO_2_, and have better tolerance to salinity, and pH, while the other lower frequency phylogroups (B2, D, E, and F) were associated with DO, temperature, and NO_3._ Our findings were consistent with Jang et al. (103) and Bong et al. (44), who reported that the occurrence and distribution of *E. coli* phylogenetic distribution can be affected by environmental variables. Collectively, a better adaptation to environmental drivers, paired with a high turnover of gene repertoires, made the phylogroup B1 exquisitely versatile in the environment compared to other phylogenetic groups (117). Our findings were in line with previous studies that nutrient concentrations (C, N, and P) are one of the important factors influencing the growth and survival of *E. coli* in the environment (104, 105). The addition of nutrient concentrations could also enhance the horizontal transfer of genetic resistance materials (106), which further enhanced the adaptive ability and plasticity of *E. coli* in a variety of environments. Lye et al. (29) detected a positive correlation between PO_4_ with sulfonamide-resistant heterotrophic bacteria and sulfonamide-enteric bacteria in Larut River. They had found that on four occasions, an exceptionally high PO_4_ concentration but low nitrogen concentration was observed in wastewater effluents from both the zoo and the hospital. However, further studies are still required to understand the nature of these anthropogenic stressors and the exact mechanisms in shaping the *E. coli* prevalence, diversity, and dissemination of antibiotic resistance in this river.

Our results also showed that the distribution of different phylogroups and antibiotic resistance genes were also affected by antibiotic use. The antibiotics detected in hospital, slaughterhouse, and S1b exhibited a significant correlation with the phylogenetic groups A, C, *sul*, and *tet* genes, while oxytetracycline (OTC) and fluoroquinolones (ENRO and ENX) detected in the zoo showed positive associations with phylogroup B1 (Figure 5B). However, no correlation was observed in the lower frequency phylogroups (B2, D, E, and F). This observation of correlations concurred with Varela et al. (107), Lye et al. (29), and Low et al. (42), who have revealed that the wastewater effluents from the zoo, slaughterhouse, and hospital are important antibiotic pollutant sources to the Larut River. Thus, the antibiotic residues in these effluents are expected to have a strong impact of selective pressure on antibiotic resistance in environmental *E. coli* compared to river waters. The variation in the response of different phylogenetic groups of *E. coli*, including resistant genes to different antibiotic residues, might be attributed to the types of antibiotics detected, physiochemical properties and persistence of antibiotics in water (64), water quality (6), acquired resistance mechanisms (108, 109), environmental fitness of *E. coli*, and indigenous microflora (58).

### Genetic Diversity of *E. coli* Through Rep-PCR Fingerprint

A total of 354 band patterns, with amplicon sizes ranging from 100 to 2,000 bp, were generated by REP-PCR. The genetic diversity of the *E. coli* population was high in effluents from the slaughterhouse (*H'*, 3.53) and the zoo (*H'*, 3.38) compared to river waters. Our observation concurred with Jang et al. (110) where diversity of *E. coli* genotypes tends to be greater with increasing proximity to anthropogenic urban sites. However, genetic heterogeneity between isolates from the natural environment may be caused by differences between the sampling sites (e.g., sampling sites were probably subjected to high pollution from various sources) (111). In contrast, hospital effluent had a low *E. coli* genetic diversity (*H'*: 2.91) among other effluent sites, even though Low et al. (42) observed elevated antibiotic concentrations. A similar observation was found by McLellan (112) who reported lower diversity of *E. coli* in contaminated surface waters, in which environmental survival may be the factor that influences the recovery of the composition of strains from contaminated waters.

**Figure 6:**
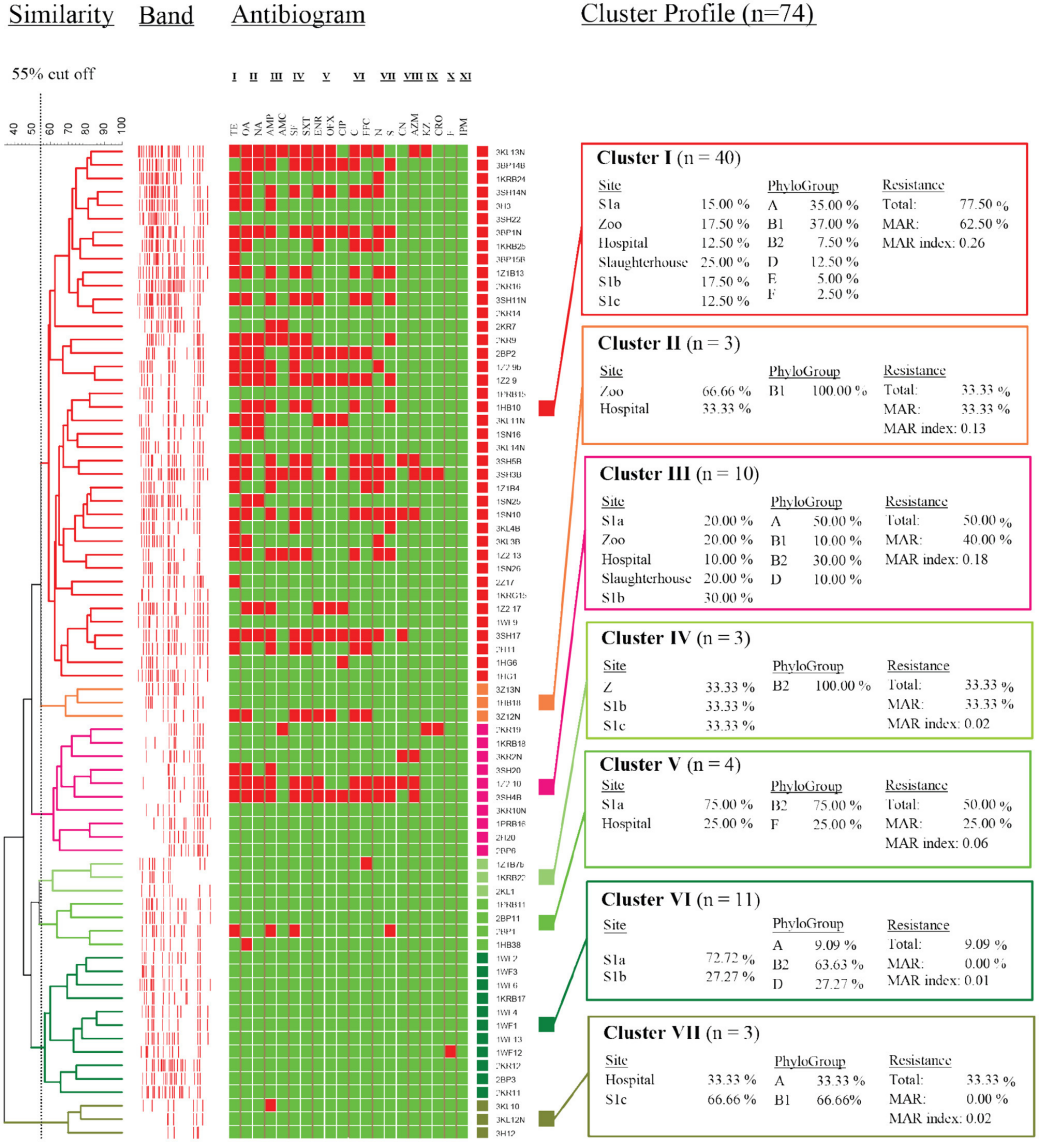
Dendrogram showing similarity of *E. coli* strains isolated from different sampling sites as determined by rep-PCR fingerprint analysis using REP primer. I, Tetracycline; II, Quinolone; III, Penicilin; IV, Sulfonamide; V, Fluoroquinolone; VI, Amphenicol; VII, Aminoglycoside; VIII, Macrolide; IX, Cephalosporin; X, Nitrofuran; XI, Carbapenem.

Due to the large population size, only the non-repeating *E. coli* genotypes (<85% similarity coefficient, *n* = 74) were selected to have their genetic relationships characterized. Seven major clusters were generated, with the highest number of isolates grouped in cluster I (*n* = 40), while the lowest number was shared among clusters II, IV, and VII (*n* = 4) (Figure 6). Cluster I isolates were found in a roughly equal distribution at all sites at Larut River, except for the hospital effluent and S1c with isolates (72%) that are characterized under the generalist phylogroups A and B1 with phenotypic resistance (77.50%). This cluster constituted the prevalence of background antibiotic resistance in this study (113–115). Similar to cluster I, cluster III (*n* = 10) were phylogroup A and B1 strains, with high antibiotic resistance, in which S1b has a higher distribution among the sites. In cluster VII, isolates from downstream site S1c co-clustered with an isolate from midstream hospital, thus, suggesting that the isolates along these sites shared a lineage. In contrast, low AR phylogroup B2 isolates were abundant in clusters IV and VI. These clusters collectively contained isolates (*n* = 14) that were located at non-anthropogenic sites, except a strain from the zoo. Similar to Liang et al. (116), there was no clear pattern of *E. coli* clustering according to sites in this study. It is important to note that the main aim of this study is not to trace the exact host sources of commensal *E. coli* in a complex aquatic environment. The diversity of *E. coli* genotypic and phenotypic is very large. According to literature, a collection of more than 20,000 isolates had only captured 27% of the predicted genotypes as estimated by a rarefaction analysis (46). This was evidenced by the hospital and the zoo isolates consisting of a diverse population with five and four different clades, respectively, which were also observed by Ghaderpour et al. (27). Given the limitation, this study did, however, demonstrate that AR *E. coli* population diversity in riverine estuarine water was, instead, related to the proximity of source contamination, stream order, and land use as observed by other studies (46, 76).

## Conclusion

The present study affirmed that the prevalence and the diversification of antibiotic-resistant *E. coli* in Larut River were intensified by wastewater effluent from zoo, hospital, and slaughterhouse as sources of antibiotic residues. Our findings showed that phylogroups B1 and A were predominant with the presence of resistance genes. The cluster analysis revealed that the antibiotic resistance phenotype distribution of *E. coli* isolates from the zoo and the slaughterhouse effluents were more similar than the hospital effluent and downstream site (S1b). The *tet* efflux genes were detected in the majority of the *E. coli* isolates, thus, suggesting that *E. coli* may be an important carrier and/or reservoir of tetracycline resistance genes conferring resistance. The prevalence of the *sul3* gene in *E. coli* isolates might be attributed to the consumption of sulfonamide for humans and veterinary use. The CCA analysis revealed a significant association between phylogroup and resistance genes with physicochemical properties and antibiotic residues on the environmental persistence of antibiotic-resistant *E. coli*. All these findings are important to provide information on the global comparison of persistence of antibiotic-resistant *E. coli* in different aquatic ecosystems, and the need to have surveillance and monitoring of virulence and antibiotic resistance in fresh river water to mitigate the emerging resistance and dissemination through water and environment.

## Data Availability Statement

The original contributions presented in the study are included in the article/Supplementary Material, further inquiries can be directed to the corresponding author/s.

## Author Contributions

CB: contributed to conceptualization, methodology, writing, and editing. KL: contributed to methodology and editing. LC: contributed to conceptualization and editing. CL: contributed to conceptualization, editing, and funding. All authors contributed to the article and approved the submitted version.

## Funding

This research was supported by the Ministry of Higher Education Malaysia under the Higher Institution Centre of Excellence (HICoE) Programme (IOES-2014D), University of Malaya (RU009D-2015), and private funding (PV009-2019) awarded to LC.

## Conflict of Interest

The authors declare that the research was conducted in the absence of any commercial or financial relationships that could be construed as a potential conflict of interest.

## Publisher's Note

All claims expressed in this article are solely those of the authors and do not necessarily represent those of their affiliated organizations, or those of the publisher, the editors and the reviewers. Any product that may be evaluated in this article, or claim that may be made by its manufacturer, is not guaranteed or endorsed by the publisher.
