# Supplementary material for: Prevalence and Diversity of Antibiotic Resistant Escherichia coli From Anthropogenic-Impacted Larut River
Source: Front Public Health. 2022 Mar 10;10:794513. doi: 10.3389/fpubh.2022.794513 (PMC8960044; doi:10.3389/fpubh.2022.794513)
Supplement: Supplementary file 1 [file Table_1.DOCX]

**SUPPLEMENTARY 1 |** List of PCR primer sequences and conditions.

|  | Target | Primer sequences (5′-3′) | Size (bp) | PCR Condition | | | Reference |
| --- | --- | --- | --- | --- | --- | --- | --- |
| ﻿*E. coli* detection | *phoA* | phoA-F  GTGACAAAAGCCCGGACACCATAAATGCCT  phoA-R  TACACTGTCATTACGTTGCGGATTTGGCGT | 903 | 94°C 2min  94°C 1min  56°C 1min  72°C 1min  72°C 10min | } | 35 cycles | Kong et al.1999 |
| Pathotyping | VT | VT-F  GAGCGAAATAATTTATATGTG  VT-R  TGATGATGGCAATTCAGTAT | 518 | 4°C 2min  92°C 30sec  59°C 30sec  72°C 30sec  72°C 5min | } | 40 cycles | Aranda et al. 2007 |
|  | *eae* | eae-F  CTGAACGGCGATTACGCGAA  eae-R  CGAGACGATACGATCCAG | 917 |  |  |  |  |
|  | *bfpA* | bfpA-F  AATGGTGCTTGCGCTTGCTGC  bfpA-R  GCCGCTTTATCCAACCTGGTA | 326 |  |  |  |  |
|  | *aggR* | aggR-F  GTATACACAAAAGAAGGAAGC  aggR-R  ACAGAATCGTCAGCATCAGC | 254 |  |  |  |  |
|  | LT | LT-F  GCACACGGAGCTCCTCAGTC  LT-R  TCCTTCATCCTTTCAATGGCTTT | 218 |  |  |  | Vidal et al. 2005 |
|  | *daaE* | daaE-F  GAACGTTGGTTAATGTGGGGTAA  daaE-R  TATTCACCGGTCGGTTATCAGT | 542 |  |  |  |  |
|  | *virF* | virF-F  CTCGGCACGTTTTAATAGTCTGG  virF-R  GTGGAGAGCTGAAGTTTCTCTGC | 618 |  |  |  |  |
|  | *ipaH* | ipaH-F  CTCGGCACGTTTTAATAGTCTGG  ipaH-R  GTGGAGAGCTGAAGTTTCTCTGC | 933 |  |  |  |  |
|  | ST | ST-F  GCTAAACCAGTAGAGCTCTTCAAAA  ST-R  CCCGGTACAGAGCAGGATTACAACA | 147 |  |  |  | Nguyen et al. 2005 |
| Phylo-grouping | *arpA* | AceK.f  AACGCTATTCGCCAGCTTGC  ArpA1.r  TCTCCCCATACCGTACGCTA | 400 | 94°C 4min  94°C 5sec  59°C 20sec  72°C 5min | } | 30 cycles | Clermont et al. 2013 |
|  | *chuA* | chuA.1b  ATGGTACCGGACGAACCAAC  chuA.2  TGCCGCCAGTACCAAAGACA | 288 |  |  |  |  |
|  | *yjaA* | yjaA.1b  CAAACGTGAAGTGTCAGGAG  yjaA.2b  AATGCGTTCCTCAACCTGTG | 211 |  |  |  |  |
|  | TspE4.C2 | TspE4C2.1b  CACTATTCGTAAGGTCATCC  TspE4C2.2b  AGTTTATCGCTGCGGGTCGC | 152 |  |  |  |  |
|  | *ibeA* | ibeA10.f  AGGCAGGTGTGCGCCGCGTAC  ibeA10.r  TGGTGCTCCGGCAAACCATGC | 170 | 95°C 12min  94°C 30sec  63°C 30sec  68°C 3min  72°C 10min | } | 25 cycles | Johnson and Stell, 2000 |
|  | *trpA*  (C-specific) | trpAgpC.1  AGTTTTATGCCCAGTGCGAG  trpAgpC.2  TCTGCGCCGGTCACGCCC | 219 | 94°C 4min  94°C 5sec  59°C 20sec  72°C 5min | } | 30 cycles | Lescat et al. 2009 |
|  | *arpA*  (E-specific) | ArpAgpE.f  GATTCCATCTTGTCAAAATATGCC  ArpAGGPe.r  GAAAAGAAAAAGAATTCCCAAGAG | 301 |  |  |  |  |
|  | *trpA*  (Internal control) | trpBA.f  CGGCGATAAAGACATCTTCAC  trpBA.r  GCAACGCGGCCTGGCGGAAG | 489 |  |  |  | Clermont et al. 2008 |
|  | *aes* (cryptic clade I) | aesl.1  CCTCTACTCACCCAAAAGTC  aesl.2  ATCACGTAACCACAACGCAC | 315 | 94°C 4min  94°C 5sec  63°C 30sec  72°C 5min | } | 30 cycles | Clermont et al. 2011 |
|  | *aes* (cryptic clade II) | aesII.1 CGCCTGTTGTCACTTCCACG  aesII.2  GTTTATCACGCAGCCACAAG | 125 |  |  |  |  |
|  | *chuA* (cryptic clade III) | chuIII.1  GTGTTGAGATTGTCCGTGGG  chuIII.2  CAAAAGCACTGGCGCCCAG | 183 |  |  |  |  |
|  | *chuA*  (cryptic clade IV) | chuIV.1  CTGGCGAAAGGAACCTGGA  chuIV.2  GTTATCTCATCTTGCAGCCAA | 461 |  |  |  |  |
|  | *chuA*  (cryptic clade V) | chuV.1  ACTGTATGGCAGTGGCGCAT  chuV.2  GCAAAACTATCGGCAAACAGC | 600 |  |  |  |  |
| Tetra-cycline Resistance | *tet*(*A*) | tetA.f  GCTACATCCTGCTTGCCTTC  tetA.r  CATAGATCGCCGTGAAGAGG | 210 | 94°C 5min  94°C 1min  65°C 1min  72°C 1min  72°C 10min | } | 35 cycles | Ng et al. 2001 |
|  | *tet*(*B*) | tetB.f  TTGGTTAGGGGCAAGTTTTG  tetB.r  GTAATGGGCCAATAACACCG | 659 |  |  |  |  |
|  | *tet*(*C*) | tetC.f  CTTGAGAGCCTTCAACCCAG  tetC.r  ATGGTCGTCATCTACCTGCC | 418 |  |  |  |  |
|  | *tet*(*D*) | tetD.f  AAACCATTACGGCATTCTGC  tetD.r  GACCGGATACACCATCCATC | 787 |  |  |  |  |
|  | *tet*(*E*) | tetE.f  AAACCACATCCTCCATACGC  tetE.r  AAAAGGCCACAACCGTCAG | 278 |  |  |  |  |
|  | *tet*(*G*) | tetG.f  CAGCTTTCGGATTCTTACGG  tetG.r  GATTGGTGAGGCTCGTTAGC | 844 |  |  |  |  |
|  | *tet*(*K*) | tetK.f  TCGATAGGAACAGCAGTA  tetK.r  CAGCAGATCCTACTCCTT | 169 |  |  |  |  |
|  | *tet*(*L*) | tetL.f  TCGTTAGCGTGCTGTCATTC  tetL.r  GTATCCCACCAATGTAGCCG | 267 |  |  |  |  |
|  | *tet*(*M*) | tetM.f  GTGGACAAAGGTACAACGAG  tetM.r  CGGTAAAGTTCGTCACACAC | 406 |  |  |  |  |
|  | *tet*(*O*) | tetO.f  AACTTAGGCATTCTGGCTCAC  tetO.r  TCCCACTGTTCCATATCGTCA | 515 |  |  |  |  |
|  | *tet*(*S*) | tetS.f  CATAGACAAGCCGTTGACC  tetS.r  ATGTTTTTGGAACGCCAGAG | 667 |  |  |  |  |
|  | *tetA*(*P*) | tetA(P).f  CTTGGATTGCGGAAGAAGAG  tetA(P).r  ATATGCCCATTTAACCACGC | 676 |  |  |  |  |
|  | *tet*(*Q*) | tetQ.f  TTATACTTCCTCCGGCATCG  tetQ.r  ATCGGTTCGAGAATGTCCAC | 904 |  |  |  |  |
|  | *tet*(*X*) | tetX.f  CAATAATTGGTGGTGGACCC  tetX.r  TTCTTACCTTGGACATCCCG | 468 |  |  |  |  |
| Sulpho-namide Resistance | *sul1* | sul1.f  CGGCGTGGGCTACCTGAACG  sul1.r  GCCGATCGCGTGAAGTTCCG | 433 | 95°C 3min  95°C 30sec  68°C 30sec  72°C 30sec  72°C 5min | } | 35 cycles | Kozak et al. 2009 |
|  | *sul2* | sul2.f  CGGCATCGTCAACATAACCT  sul2.r  TGTGCGGATGAAGTCAGCTC | 721 |  |  |  |  |
|  | *sul3* | sul3.f  TCCGTTCAGCGAATTGGTGCAG  sul3.r  TTCGTTCACGCCTTACACCAGC | 128 | 95°C 3min  95°C 30sec  64°C 30sec  72°C 30sec  72°C 5min | } | 35 cycles | Pei et al. 2006 |
| Genetic Diversity | REP | REP  GCGCCGICATGCGGCATT |  | 94°C 7min  94°C 30sec  47°C 1min  72°C 1min  72°C 4min | } | 35 cycles | Lim et al. 2009 |
